# A transferrin receptor targeting dual-modal MR/NIR fluorescent imaging probe for glioblastoma diagnosis

**DOI:** 10.1093/rb/rbae015

**Published:** 2024-02-17

**Authors:** Jiaqi Hao, Huawei Cai, Lei Gu, Yiqi Ma, Yan Li, Beibei Liu, Hongyan Zhu, Fanxin Zeng, Min Wu

**Affiliations:** Department of Radiology and Huaxi MR Research Center (HMRRC), Functional and Molecular Imaging Key Laboratory of Sichuan Province, West China Hospital, Sichuan University, Chengdu, Sichuan 610041, China; Research Unit of Psychoradiology, Chinese Academy of Medical Sciences, Chengdu, Sichuan 610041, China; Department of Nuclear Medicine & Laboratory of Clinical Nuclear Medicine, West China Hospital, Sichuan University, Chengdu, Sichuan 610041, China; Department of Radiology and Huaxi MR Research Center (HMRRC), Functional and Molecular Imaging Key Laboratory of Sichuan Province, West China Hospital, Sichuan University, Chengdu, Sichuan 610041, China; Department of Radiology and Huaxi MR Research Center (HMRRC), Functional and Molecular Imaging Key Laboratory of Sichuan Province, West China Hospital, Sichuan University, Chengdu, Sichuan 610041, China; Department of Radiology and Huaxi MR Research Center (HMRRC), Functional and Molecular Imaging Key Laboratory of Sichuan Province, West China Hospital, Sichuan University, Chengdu, Sichuan 610041, China; Department of Radiology and Huaxi MR Research Center (HMRRC), Functional and Molecular Imaging Key Laboratory of Sichuan Province, West China Hospital, Sichuan University, Chengdu, Sichuan 610041, China; State Key Laboratory of Biotherapy, West China Hospital, Sichuan University, Chengdu, Sichuan 610041, China; Department of Clinical Research Center, Dazhou Central Hospital, Dazhou, Sichuan 635000, China; Department of Radiology and Huaxi MR Research Center (HMRRC), Functional and Molecular Imaging Key Laboratory of Sichuan Province, West China Hospital, Sichuan University, Chengdu, Sichuan 610041, China; Research Unit of Psychoradiology, Chinese Academy of Medical Sciences, Chengdu, Sichuan 610041, China

**Keywords:** glioblastoma, magnetic resonance imaging, dual-modal probes, near-infrared fluorescent, targeting peptides

## Abstract

The prognosis of glioblastoma (GBM) remains challenging, primarily due to the lack of a precise, effective imaging technique for comprehensively characterization. Addressing GBM diagnostic challenges, our study introduces an innovative dual-modal imaging that merges near-infrared (NIR) fluorescent imaging with magnetic resonance imaging (MRI). This method employs superparamagnetic iron oxide nanoparticles coated with NIR fluorescent dyes, specifically Cyanine 7, and targeted peptides. This synthetic probe facilitates MRI functionality through superparamagnetic iron oxide nanoparticles, provides NIR imaging capability via Cyanine 7 and enhances tumor targeting trough peptide interactions, offering a comprehensive diagnostic tool for GBM. Notably, the probe traverses the blood–brain barrier, targeting GBM *in vivo* via peptides, producing clear and discernible images in both modalities. Cytotoxicity and histopathology assessments confirm the probe’s favorable safety profile. These findings suggest that the dual-modal MR\NIR fluorescent imaging probe could revolutionize GBM prognosis and survival rates, which can also be extended to other tumors type.

## Graphical abstract

**Figure:**
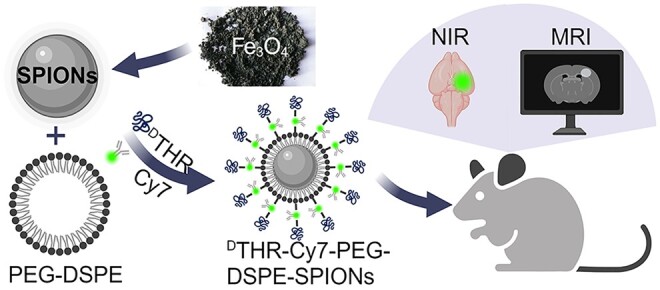

## Introduction

Glioblastoma (GBM) is the prevailing primary malignant brain tumor, constituting nearly 16% of all primary brain tumors [1]. The standard treatment for GBM includes surgical resection that minimizes damage to healthy brain tissue, combined with chemotherapy and radiotherapy [2, 3]. However, the prognosis of GBM remains poor due to its high recurrence and metastasis rates. This is attributed to: (i) GBM’s diffuse growth making complete resection challenging [4]; (ii) the need to preserve critical brain areas limiting surgical options [5]; and (iii) the insensitivity of residual tumor tissues to radiotherapy and chemotherapy [6]. Consequently, accurate delineation of GBM’s growth range and tumor margins can enable more effective surgical resections, potentially improving prognosis.

Presently, the primary diagnostic modalities for GBM each have distinct pros and cons. Positron emission computed tomography (PET) and single photon emission computed tomography are sensitive to tumors, but have limited resolution and involve radioactivity, making precise tumor delineation challenging [7,8]. Computed tomography (CT) imaging is rapid and convenient but falls short in resolution and detecting small lesions [,^9^]. Optical imaging is useful for intraoperative navigation but is impeded by the skull’s opacity [10,]. Magnetic resonance imaging (MRI) has emerged as a cornerstone due to its remarkable advantages, which can provide excellent sub-millimeter resolution, lead to effective soft tissue contrast [11], such as effectively detecting cerebral edema, necrosis and heterogeneous ring masses in GBM [12,13]. Nonetheless, clinically used non-specific gadolinium (Gd)-based contrast agents often fail to penetrate the blood–brain barrier (BBB) and entering intracranial tumor tissue. Researches indicate that glioblastomas generally do not manifest significant BBB disruption in the initial stage [14–16]. This limitation hinders the effectiveness of these contrast agents in identifying more than 10% of GBM cases in enhanced MRI scans [17]. In addition, Gd-based MRI contrast agents pose risks of renal toxicity and potential Gd deposition in the brain [18]. Recently, clinicians are increasingly exploring multimodal imaging in neuroscience, which offers improved specificity and sensitivity by overcoming the limitations of individual modalities [19]. Therefore, developing stable and efficient tumor-targeted probes to delineate tumor boundaries pre- or intraoperatively holds significant importance.

However, single MRI diagnosis falls short for preoperative diagnosis and intraoperative navigation due to brain parenchyma distortion from factors like cerebrospinal fluid loss, edema and tumor resection. This makes preoperative MRI images unreliable for intraoperative use. In contrast, intraoperative optical imaging can provide real-time updates of the brain parenchyma, aiding in the accurate detection of residual tumor tissue. Therefore, our goal is to develop a probe that enhances GBM diagnosis sensitivity and integrates with optical imaging, facilitating both preoperative diagnosis and intraoperative navigation.

Superparamagnetic iron oxide nanoparticles (SPIONs), a class of paramagnetic MRI contrast agent, have received clinical approval for several decades [20–22]. SPIONs exhibit ideal GBM-specific probe properties, including high performance, biocompatibility, prolonged blood circulation time and efficient surface functionalization. BBB is a protective barrier formed by glial cells and capillary wall, separating circulating blood from brain tissue [23, 24]. Typically, the BBB utilizes specific transport systems to regulate the movement of essential nutrients and metabolites into brain tissue [25–27]. Therefore, utilizing receptor-mediated transcytosis across the BBB could be an effective strategy to transport the probes into the brain. The transferrin receptor (TfR), highly expressed at the BBB and in brain tumor tissues, is one of the most representative mediate receptors, enabling BBB penetration and tumor tissue targeting [28]. Indeed, rapid enzymatic degradation and competition with endogenous transferrin hinder the effective binding of anti-TfR antibodies or ligands to TfR, diminishing their targeting efficacy. To overcome this challenge and enhance stability, peptides are specifically designed to align with the tumor’s microenvironment and specific requirements [29–31].

In this study, we constructed a ligand-guided nanoprobe with high affinity and high stability based on the multi-modal imaging strategy. First, we employed SPIONs as the core nanoplatform, integrating them with the near-infrared (NIR) dye Cyanine 7 (Cy7) to construct an MR/NIR dual-modal nanoprobe. THR peptide (Ac-THRPPMWSPVWP-COOH, a TfR ligand), facilitated receptor-mediated endocytosis, aiding the nanoprobes’ BBB penetration and specific GBM imaging [32, 33]. We enhanced THR’s *in vivo* stability by using its reverse enantiomer ^D^THR (Ac-pwvpswmpprht-COOH), to resist enzymatic degradation in the bloodstream [34]. Systematic evaluation of the probes’ BBB transport and GBM targeting in intracranial mouse models yielded favorable results. The study highlights this nanoprobe’s potential in dual-mode MRI/NIR fluorescence imaging for GBM, enhancing preoperative diagnosis and intraoperative navigation.

## Materials and methods

### Materials

DSPE-PEG(2000)-amine and phosphate buffer solution (PBS) were purchased from Sigma-Aldrich. The luciferase-plasmid was supplied by the State Key Laboratory of Biotherapy (Chengdu, China). Cy7 was offered by Professor Bowen Ke from the Department of Anesthesiology at West China Hospital, Sichuan University. DOTA-^D^THR was obtained from ChinaPeptides.

### Synthesis and characterization of nanoprobes

We used the standard solid-phase synthesis method to synthesize THR (Ac- THRPPMWSPVWP-OH) and ^D^THR (Ac-pwvpswmpprht-OH), then purified them by high-performance liquid chromatography. SPIONs were synthesized utilizing the conventional hydrothermal technique. First, iron acetone, 1, 2-diol, oleic acid, oleamide and benzyl were mixed, and the air was expelled from the reaction system with an inert gas. Then, heated 2 h at 200°C, followed by heating 1 h at 300°C, the reactant turns black. Let cool to room temperature and add ethanol to precipitate. Then, centrifugation removes the liquid and precipitates dispersed in hexane. Eventually, the gained SPIONs solution was centrifuged and aggregates are extracted, and the SPIONs were coated with DSPE-PEG(2000)-amine. The successfully synthesized NH_2_-PEG-DSPE-SPIONs were preprocessed in the 0.02 M pH = 8 borate buffer through the ultrafiltration tube. Simultaneously, we dissolved 2 mg ^D^THR into 0.02 M MES buffer (pH = 5.5), then mixed with 1 mg N-(3-dimethylaminopropyl)-N-ethyl-carbodiimide hydrochloride (EDC, Sigma-Aldrich) and 0.5 mg N-hydroxysuccinimide (NHS, Sigma-Aldrich) to activate the carbonyl. The residual EDC and NHS were removed using 2 kDa ultrafiltration tubes via centrifugation at 3000 rpm. Subsequently, the activated peptides were mixed with the NH_2_-PEG-DSPE/SPIONs solution on a shaker for 2 h. The ^D^THR-PEG-DSPE-SPIONs were isolated using centrifugal ultrafiltration, and then resuspended in PBS buffer. Finally, 50 *µ*L 1 mg/mL Cy7-NHS was added to the solution, stirred overnight at 4°C in the dark and then ultrafiltered to remove unreacted dye. The resulting ^D^THR-Cy7-PEG-DSPE-SPIONs probes were immediately stored at 4°C.

An ultraviolet spectrophotometer (UV3600, SHIMADZU) was used to quantify the grafting ratio of the ^D^THR peptide. A microplate reader (BioTek, Synergy Mx) determined the binding efficiency of NIR dyes to SPIONs. Transmission electron microscopy (TEM), selected area electron diffraction and dynamic light scattering (DLS) were used to characterize the morphology, electrical properties and size distribution of the probes. An MPMS7 Quantum Design SQUID magnetometer with an applied field ranging from −3.0 to 3.0 T at 300 K was used to measure the magnetic properties (Quantum Design, San Diego, USA). MRI were obtained using a 3.0 T MR (Siemens TrioTim) at room temperature. The *T*_2_ relaxation times were calculated using the formula 1/*T*_2_=1/*T*_2_^0^+*r*_2_ C_Fe_, where 1/*T*_2_^0^ represents the relaxation rate of water without a probe, and C_Fe_ indicates the concentration of Fe.

In order to detect the biological metabolism of the probe, we synthesized ^68^Ga-DOTA-^D^THR. Chelation of ^68^Ga with sodium acetate was performed after adjusting the pH to four. Then, the reaction mixture was heated at 95°C for 10 min, and its completion was verified using radio-liquid chromatography. Finally, ^68^Ga-DOTA-^D^THR underwent solid-phase extraction before being utilized in PET imaging.

### Assay of cytotoxicity

HUVEC, bEnd.3 cells and U87-MG cells were obtained from the Chinese Academy of Medical Sciences. High-glucose DMEM medium (Hyclone), supplemented with 10% fetal bovine serum (Prime) and 1% penicillin/streptomycin (Hyclone), was used to maintain all cells under the 5% CO_2_ at 37°C. HUVEC and U87-MG cells were seeded at a density of 1 × 10^4^ cells/well in 96-well plates, then incubated overnight at 37°C in a 5% CO_2_ environment. Finally, the cells were treated with media containing probes at varying Fe concentrations (0, 3.125, 6.25, 12.5, 25 and 50 *µ*g/mL). Following cultivation 24 h, cells were exposed to a mixture of 10 *µ*L CCK-8 with 90 *µ*L DMEM for 2 h, followed by absorbance measurement at 450 nm.

### Toxicity test *in vivo*

For *in vivo* toxicity assessment, healthy BALB/c male mice (4–6 weeks) received a single-dose of 10 mg Fe_3_O_4_/kg (targeted/untargeted nanoprobes) or an equivalent volume of saline via the tail vein. Three mice from each group were euthanized at predetermined time points (1, 3 and 7 days) post-injection. Subsequently, the spleen, heart, kidney, liver and lung tissues were then excised, fixed, embedded, sectioned and stained with H&E. Histological changes were observed by a light microscope.

### Stability essay of peptides

To assess the *in vivo* stability of the peptide, PET/CT imaging was performed using the IRIS small animal PET/CT system (Inviscan SAS, Strasbourg, France). Each mouse was intravenously injected with 3.7 MBq of ^68^Ga-DOTA-^D^THR and imaged an hour post-injection. PET data were acquired over 10 min and reconstructed using a 3D ordered-subset expectation–maximization algorithm with a Monte Carlo model. CT scans were conducted at sets of 1 mA, 50 kV and a duration of 140 s.

### Cellular internalization

Cell labeling efficiency and intracellular distribution of ^D^THR-Cy7-PEG-DSPE-SPIONs were respectively assessed using flow cytometry (BD FACSCelesta™) and NIR fluorescence imaging microscopy (Carl Zeiss, Axio Observer A1). U87-MG cells were inoculated in a 12-well plate at a density of 1 × 10^5^ cells per well. The adherent cells were treated with 50 *µ*g Fe_3_O_4_/mL ^D^THR-Cy7-PEG-DSPE-SPIONs for different incubation times (1, 2 and 4 h). The control group consisted of cells without probe labeling. After incubation, cells were washed and resuspended in 300 *µ*L of PBS, followed by flow cytometry analysis using the APC-A750 signal channel. And the data were analyzed with Flowjo.

To examine the stability of ^D^THR peptide and the effect of the serum on receptor–ligand interactions, targeted probes were pre-incubated with human serum for 3 h at 37°C to obtain pretreated ^D^THR-Cy7-PEG-DSPE-SPIONs. bEnd.3 and U87-MG cells were seeded in a 24-well plate at 2.5 × 10^4^ cells per well. Post-adhesion, cells were treated with ^D^THR-Cy7-PEG-DSPE-SPIONs and pretreated ^D^THR-Cy7-PEG-DSPE-SPIONs in DMEM (50 *µ*g/mL Fe_3_O_4_) for additional 2 or 4 h. Cells were washed, fixed with 4% paraformaldehyde (PFA) in PBS, then stained by 4',6-diamidino-2-phenylindole (DAPI).

### Construction of BBB model *in vitro*

A primary BBB model using bEnd.3 cells *in vitro* was established to evaluate the penetration ability of ^D^THR-Cy7-PEG-DSPE-SPIONs. The inner side of a polycarbonate transwell (Millipore, USA) with a mean pore size of 3 *µ*m and a surface area of 0.33 cm^2^ was coated with collagen. After coating, the transwell was left to air dry for 30 min. Then, bEnd.3 cells (5 × 10^4^ cells per well) were seeded into the upper chamber of a 24-well transwell plate. After 48 h, 1 × 10^5^ U87-MG cells were seeded in the lower chamber of each well. Both cells were co-cultured for 1 week.

BBB integrity was assessed using a water-leaking test and immunofluorescence to determine its permeability. Two hundred microliter and 900 *µ*L of medium were added to the upper and lower chambers, respectively. Fluid levels in each chamber were recorded at the beginning and after 4 h period. For the *in vitro* BBB model preparation, bEnd.3 cells were fixed in 4% PFA for 30 min, then washed with PBS twice. Subsequently, bEnd.3 cells were incubated in PBS containing 3% BSA for 1 h, followed by incubation with a rabbit polyclonal antibody against ZO-1 and FITC labeled anti-rabbit IgG (Invitrogen). Finally, the fixed cells were stained with DAPI for 10 min and observed under a confocal laser scanning microscope (Nikon, A1RMP+).

The endothelial permeability coefficient of sodium fluorescein (NaFl) was used to evaluate the function of BBB model *in vitro,* as previously described [32]. In brief, 200 *µ*L NaFl solution (10 *µ*g/mL) and 900 *µ*L PBS were added to the upper and lower chambers of the BBB models, respectively. And then cultured in a 5% CO_2_ atmosphere at 37°C. Following incubation periods of 30, 60 and 90 min, 100 *µ*L samples from the lower chambers were transferred to a new 96-well plate. Meanwhile, an equivalent volume of PBS was added to the lower chamber to maintain consistent volume levels. Finally, the relative fluorescence intensity of the samples was measured using a multi-detector monochromator microplate reader (485/535 nm), with NaFl concentration deduced from a standard curve. The apparent permeability coefficient (Papp) was calculated using the formula:

$$\text{Papp}=dQ/dt\times 1/\left(A\times C_{0}\right).$$

In this context, (d*Q*/d*t*) represents the transfer rate or flux of NaFl across the membrane. Here, ‘*A*’ denotes the surface area of the membrane and ‘*C*_0_’ refers to the initial concentration of NaFl within the chamber. This parameterization is crucial for understanding the diffusion characteristics relevant to MRI contrast agent.

### BBB penetrability of the probes

Two hundred microliters of either Cy7-PEG-DSPE-SPIONs solution or ^D^THR-Cy7-PEG-DSPE-SPIONs solution (10.0 *µ*g/mL Fe_3_O_4_ concentration) was introduced into the upper chamber of BBB model. Meanwhile, 900 *µ*L PBS was added to the lower chamber of the transwell container. After a 60-min incubation, 100 *µ*L of this solution was extracted to analyze the BBB permeability of nanoprobes through quantification of fluorescence intensity.

### Construction of GBM model *in situ*

Male BALB/c nude mice aged 5–7 weeks were purchased from the Animal Research Institute, Sichuan Academy of Medical Sciences, Chengdu, China, and housed in a controlled, specific pathogen-free environment. Luciferase plasmids were introduced into U87-MG cells through lentiviral vector. The successfully transfected cells, named Luc-U87-MG, were identified by their luciferase activity using an *in vivo* imaging system (IVIS) spectrum instrument. Subsequently, 5 × 10^4^ Luc-U87-MG cells were surgically implanted into the brain of each mouse. Basically, we peeled the skin off the anesthetized mouse, exposed the skull and drilled a small hole into the skull on the right side of the brain. After fixing the insertion depth of the needle, 5 *µ*L of single cell suspension was injected into the brain tissue carefully, and the needle was slowly pulled out after 5 min. Finally, the skull is sealed with bone wax and the skin is sutured. Post-implantation, tumor growth was systematically monitored over 3 weeks using an IVIS. All animal experiments were conducted following ethical approval from the Animal Care and Use Committee of Sichuan University (2018116A).

### *In vivo* MRI studies

MRI scans were performed on a clinical 3.0 T magnetic resonance scanner, employing the following imaging parameters for *T_2_*-weighted imaging (*T*_2_WI):

$$\begin{array}{c} \text{TR}=3000\;\text{ms};\;\text{TE}=93\;\text{ms};\;\\ \text{Field of vision}\;\left(\text{FOV}\right)=66\;\text{mm}\times 66\;\text{mm};\;\\ \text{Slice thickness}\;\left(\text{SL}\right)=1.0\;\text{mm};\;\\ \text{Matrix}=256\times 256;\;\text{NEX}:\;5. \end{array}$$

Tumor-bearing mice were divided into two groups (*n* = 4 per group) randomly. *T*_2_WI images of GBM tumor-bearing mice were acquired at different time points (0, 2, 4 and 24 h) post-intravenous administration of Cy7-PEG-DSPE-SPIONs or ^D^THR-Cy7-PEG-DSPE-SPIONs probes each at a dose of 10 mg Fe_3_O_4_ per kg.

### *In vivo* NIR fluorescence imaging of GBM

After MRI, both groups of mice received an intraperitoneal injection of luciferin sylvite at a dosage of 150 mg/kg. Twenty-four hours later, mice were sacrificed under anesthesia via intracardiac perfusion, followed by fixation with 4% formaldehyde. Then, the brain and major organs were extracted to demonstrate efficiency of GBM targeting and the distribution of probes in the organs was observed by an IVIS^®^ Spectrum with excitation at 720 nm and emission at 820 nm. After NIR fluorescence imaging, the isolated mouse brains underwent histological analysis with H&E and Prussian blue staining.

### Probes distribution of mice

The content of iron in the major organs of tumor-bearing mice was determined by coupled plasma mass spectrometry (ICP-MS) (VG PQExCell, TJA, USA) 24 h post-injection of the nanoprobes. BALB/c mice were divided into three groups, the experimental groups receiving 10 mg Fe_3_O_4_/kg of either Cy7-PEG-DSPE-SPIONs or ^D^THR-Cy7-PEG-DSPE-SPIONs, and a control group receiving a 0.9% sodium chloride solution. All animals were euthanized 24 h post-injection, and their major organs were excised, weighed and fully digested using 1 mL hydrogen peroxide and 2 mL nitric acid.

### Statistical analysis

All data analyses were deemed statistical significance at *P *<* *0.05. The data are expressed as means ± standard deviation. The data were analyzed by ANOVA and Student’s two-tailed *t*-test.

## Results and discussion

### Synthesis and characterization of ^D^THR-Cy7-PEG-DSPE-SPIONs

Nanoprobes were synthesized following an improved protocol. Monodispersed SPIONs nanoparticles were synthesized using the organic thermal decomposition method, subsequently encapsulated with amphiphilic DSPE-PEG. Due to its unique hydrophilic–hydrophobic segments and self-assembly properties, DSPE-PEG conferred colloidal stability to the SPIONs in circulation and facilitate their monodispersity in aqueous solutions, thus achieving better stability and biocompatibility. Subsequently, ^D^THR and Cy7 dyes were then conjugated to the PEG-coated SPIONs, forming multifunctional targeting nanoprobes.

**Figure 1.**
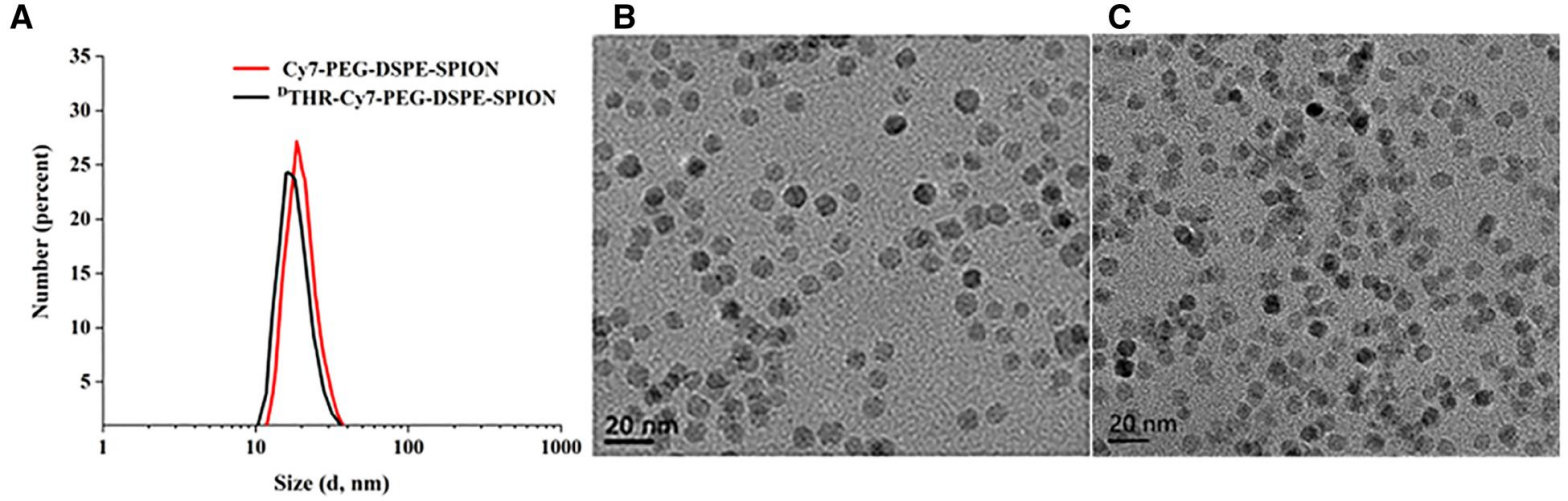
(**A**) DLS measurements of Cy7-PEG-DSPE-SPIONs and ^D^THR-Cy7-PEG-DSPE-SPIONs; (**B**) TEM images of Cy7-PEG-DSPE-SPIONs; and (**C**) ^D^THR-Cy7-PEG-DSPE-SPIONs.

UV–Vis spectroscopy revealed a noticeable absorption decrease in the supernatant at about 280 nm, comparing pre- and post-reaction samples. This decrease indicated a reduced amino acids presence post-coupling, implying successful ^D^THR peptide attachment to NH_2_-PEG-DSPE-SPIONs (see Supplementary Figure S1). Meanwhile, the conjugation efficiency, calculated as 77.12%, was derived from fluorescence intensity of post-reaction to original supernatant. The excitation and emission peak of ^D^THR-Cy7-PEG-DSPE-SPIONs at 720 or 820 nm, respectively, confirm successful conjugation of Cy7 by using a multifunctional fluorescence microplate reader. The amount of Cy7 bound to the probes reached 11.34 *µ*g (see Supplementary Figure S2). The size and morphology of the multifunctional nanoprobe were characterized by DLS and TEM. DLS measurements showed that both Cy7-PEG-DSPE-SPIONs and ^D^THR-Cy7-PEG-DSPE-SPIONs had a similar diameter of ∼20 ± 7 nm (Figure 1A). TEM imaging revealed that the Cy7-PEG-DSPE-SPIONs and ^D^THR-Cy7-PEG-DSPE-SPIONs possess an inorganic SPIONs core with a uniform size distribution of 10 ± 4 nm (Figure 1B and C). Notably, size measured by DLS were smaller than those determined by TEM owing to the PEGylation shell layer. Furthermore, these nanoparticles exhibited a slight negative charge, which can significantly prolong blood circulation, while the small size effect was beneficial to the BBB penetration and tumor infiltration of the SPIONs nanoparticles. Hence, ^D^THR-Cy7-PEG-DSPE-SPIONs have great potential in GBM targeted diagnosis in term of the physicochemical properties.

### Magnetic properties and *T*_2_-weighted magnetic resonance relaxometry

**Figure 2.**
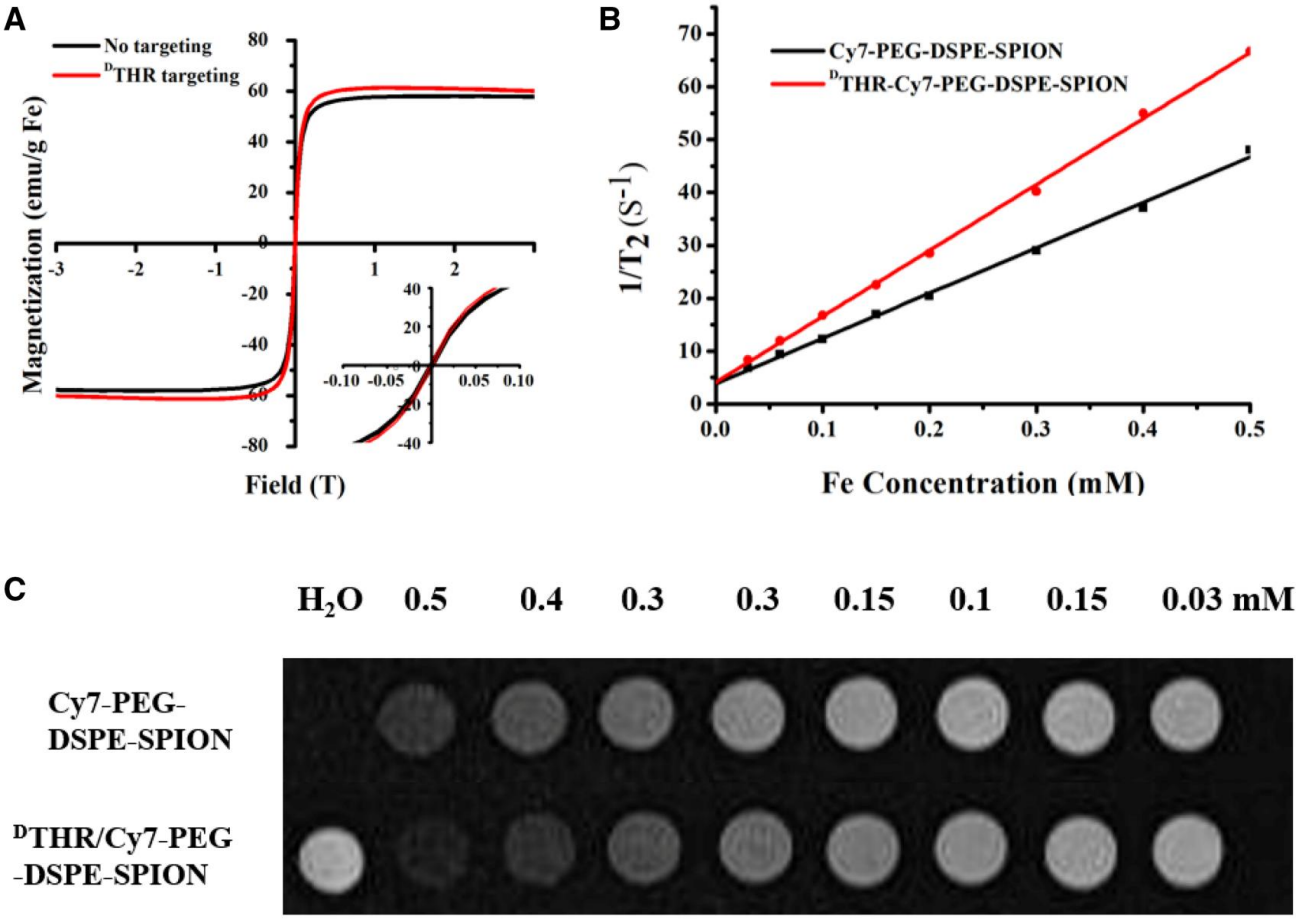
(**A**) The hysteresis loop of no targeting and ^D^THR targeting measured at 300 K; (**B**) *r_2_* values of Cy7-PEG-DSPE-SPIONs and ^D^THR-Cy7-PEG-DSPE-SPIONs probes at 3.0 T magnetic field; and (**C**) *T_2_*-weighted MR images of Cy7-PEG-DSPE-SPIONs and ^D^THR-Cy7-PEG-DSPE-SPIONs probes with different concentrations at 3.0 T magnetic field.

Hysteresis loop measurements of the nanoprobes were obtained in an external magnetic field from −3 to 3 T at 300 K (Figure 2A). Results showed that nanoprobes achieved saturation magnetization at a lower magnetic field range. Specifically, Cy7-PEG-DSPE-SPIONs exhibited a saturation magnetization of 57 emu/g Fe, and ^D^THR-Cy7-PEG-DSPE-SPIONs demonstrated 62 emu/g Fe. Moreover, magnetization values returned to zero when the magnetic field was removed. These results demonstrated that the nanoprobes exhibited superparamagnetism at room temperature, and their sensitivity to an external magnetic field.

To evaluate the effectiveness of multifunctional SPIONs as MR contrast agents, *T*_1_ and *T*_2_ relaxations of the probes were measured by a clinical 3.0 T MRI scanner. The linear regression is as follows in Figure 2B, and the *r_2_* values of Cy7-PEG-DSPE-SPIONs and ^D^THR-Cy7-PEG-DSPE-SPIONs were separately 87.2 and 121.4 S^−1 ^mM^−1^. As presented in Figure 2C, the *T*_2_ signal of multifunctional SPIONs decreases dramatically with the increasing Fe concentration. The reason for this difference may be resulted from the difference in their particle size. Despite having similar *r_1_* values, 1.8 S^−1 ^mM^−1^ for Cy7-PEG-DSPE-SPIONs and 4.1 S^−1 ^mM^−1^ for ^D^THR-Cy7-PEG-DSPE-SPIONs. And the *r_2_*–*r_1_* ratios for each type of probes were up to 30 under 3.0 T. These results suggest that their suitability for effective *T*_2_WI.

### Cytotoxicity *in vitro* and toxicity *in vivo*

**Figure 3.**
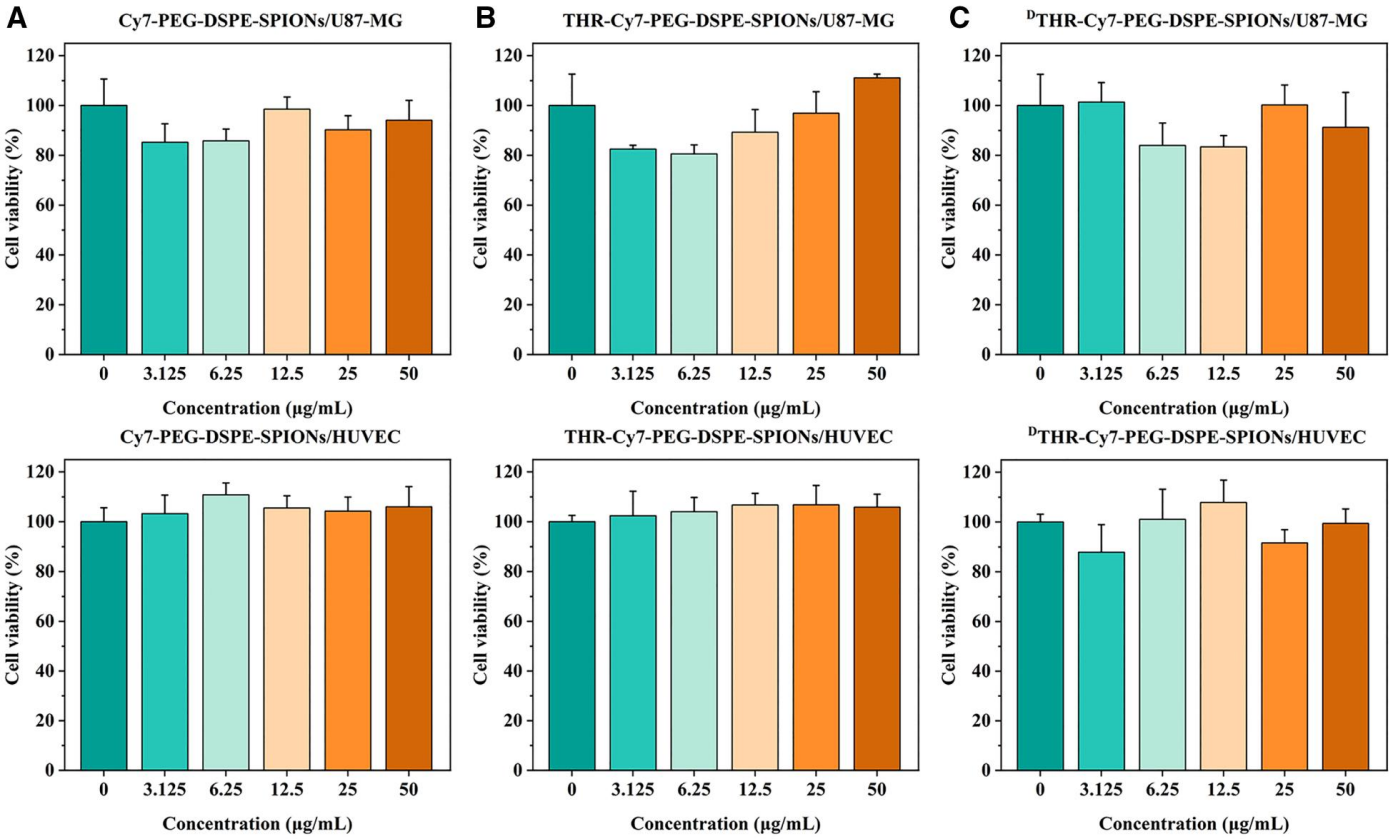
The cytotoxicity of (**A**) Cy7-PEG-DSPE-SPIONs, (**B**) THR-Cy7-PEG-DSPE-SPIONs and (**C**) ^D^THR-Cy7-PEG-DSPE-SPIONs on the HUVEC and U87-MG cells.

The proliferation rates of HUVEC and U87-MG cells can serve as benchmark for assessing cytotoxicity. CCK-8 assays showed that both cell types, when incubated with Cy7-PEG-DSPE-SPIONs, THR-Cy7-PEG-DSPE-SPIONs and ^D^THR-Cy7-PEG-DSPE-SPIONs for 24 h whose Fe_3_O_4_ concentrations ranging from 0 to 50 *µ*g/mL, exhibited no significant change in proliferation compared to the control group (Figure 3). These preliminary results suggest that the nanoprobes have good biocompatibility.

Long-term toxicity of SPIONs probes was confirmed by histological examinations *in vivo*. Healthy BALB/c mice were injected with either Cy7-PEG-DSPE-SPIONs or ^D^THR-Cy7-PEG-DSPE-SPIONs nanoprobes. At 1, 3 and 7 days post-injection, mice were sacrificed and major organs (spleen, heart, kidney, liver and lung) were harvested for H&E staining. Results indicated no significant pathological changes in all organs of the nanoprobe-injected groups compared to the control group.

### Cellular internalization

Generally, nanoparticles tend to adsorb serum molecules onto their surface forming a biological corona upon exposure to serum. This phenomenon can obscure the ligands on the nanoparticle’s surface, hindering effective recognition of target receptors and resulting in decreased cellular uptake.

**Figure 4.**
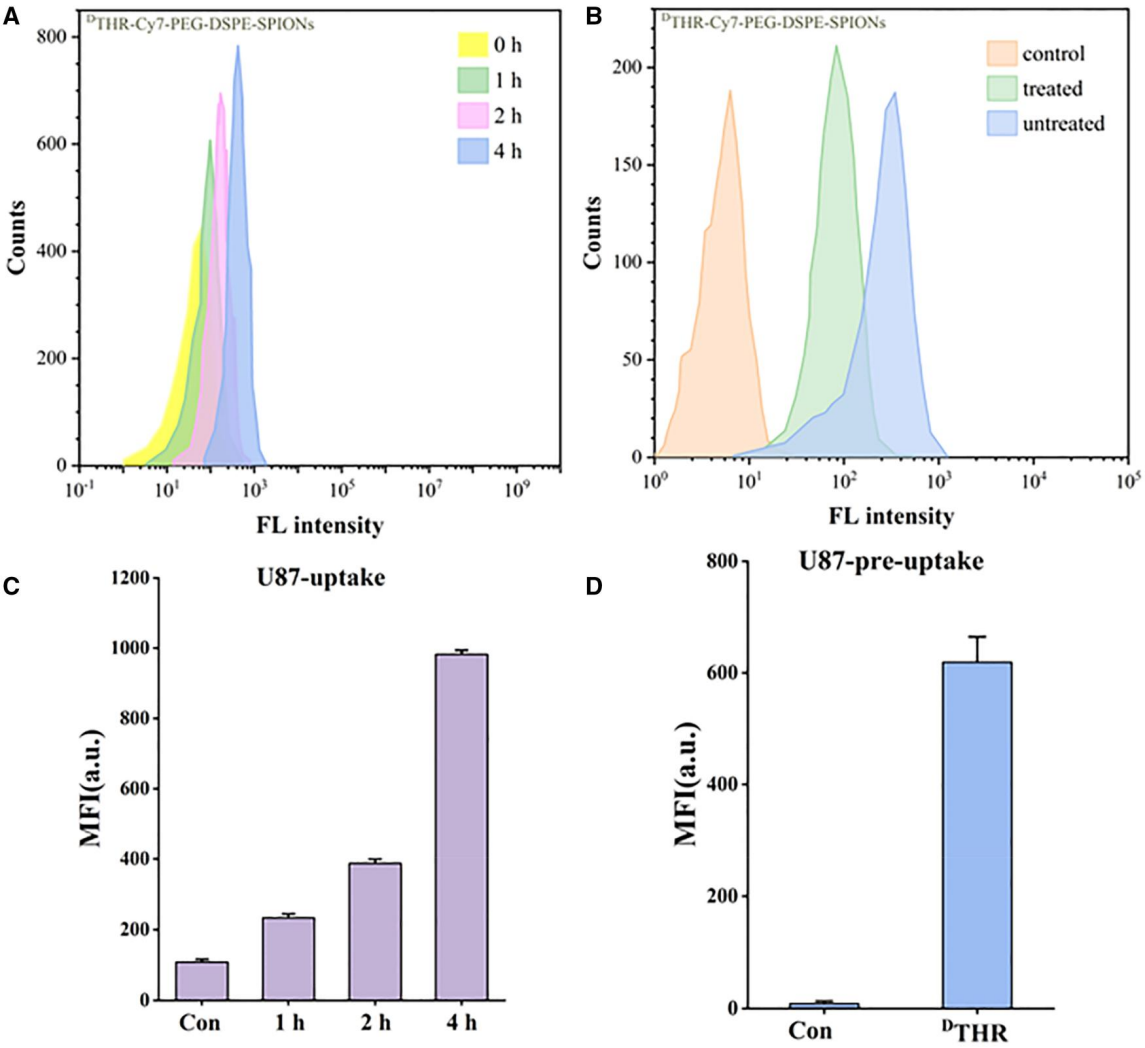
(**A**) Typical flow cytometry profiles of the cellular uptake of ^D^THR-Cy7-PEG-DSPE-SPIONs probes by U87-MG cells; (**B**) flow cytometry profiles indicating cellular uptake of the untreated/pretreated ^D^THR-Cy7-PEG-DSPE-SPIONs probes in U87-MG cells; (**C**) quantitative results of the time-dependent internalization of ^D^THR-Cy7-PEG-DSPE-SPIONs probes in U87-MG cells; and (**D**) quantitative results of cellular uptake of pre-incubated ^D^THR-Cy7-PEG-DSPE-SPIONs probes in U87-MG cells. The presented data represent the means obtained from a minimum of three independent experiments, *n* = 3, ± SEM., *P *<* *0.05.

**Figure 5.**
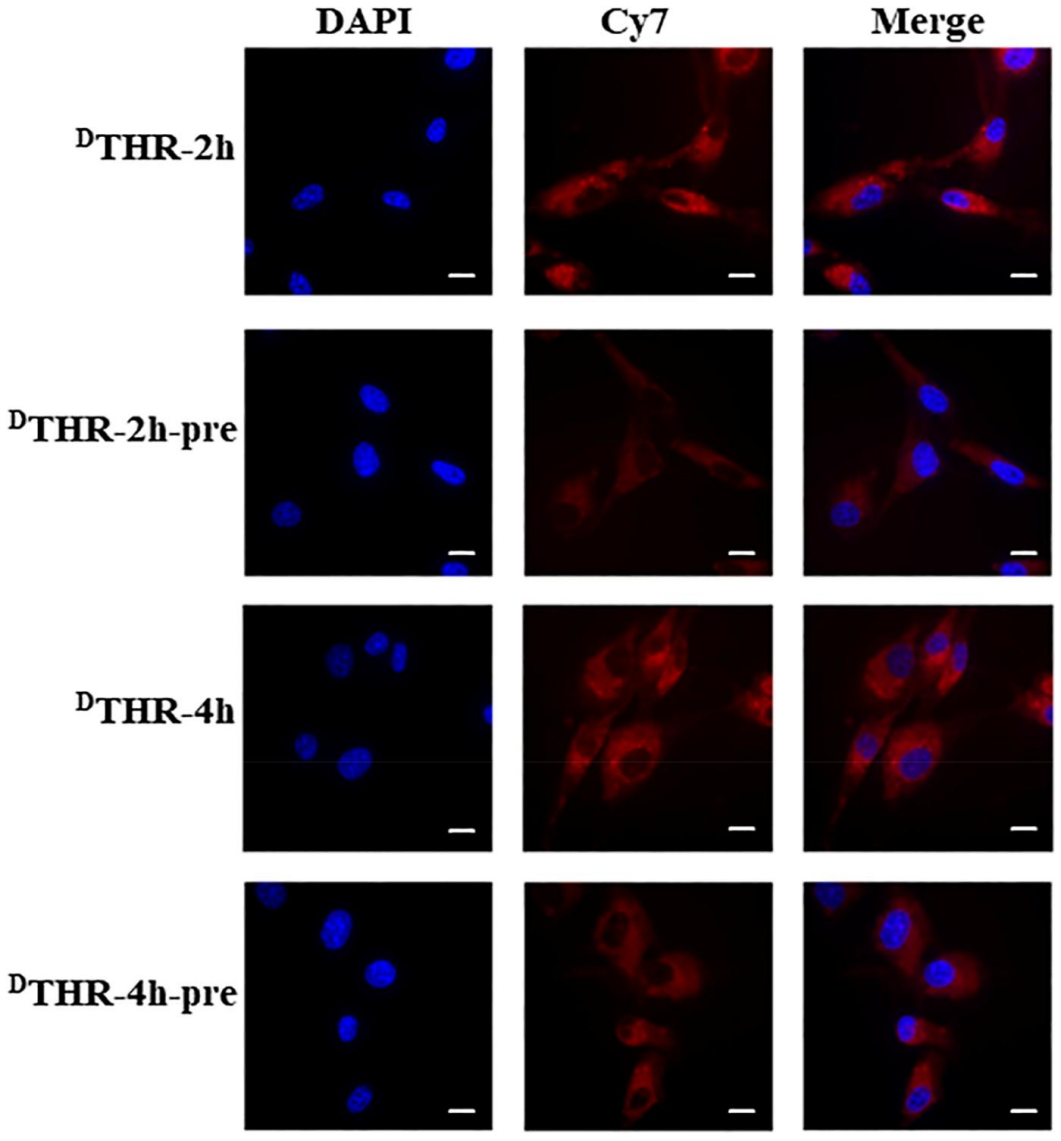
NIR fluorescence imaging demonstrates that the cellular uptake of ^D^THR-Cy7-PEG-DSPE-SPIONs probes (untreated ^D^THR) is slightly higher than that of the pretreated ^D^THR-Cy7-PEG-DSPE-SPIONs probes (treated ^D^THR) by U87-MG cells; scale bars: 25 *µ*m.

We investigated the cellular uptake of serum-pretreated ^D^THR-Cy7-PEG-DSPE-SPIONs. The binding capability of ^D^THR-modified SPIONs to TfR-expressing cells was analyzed by NIR fluorescence imaging microscopy and FCM. Results exhibited that both untreated and serum-pretreated ^D^THR-Cy7-PEG-DSPE-SPIONs were effectively internalized by U87-MG and bEnd.3 cells. Moreover, the uptake of ^D^THR-Cy7-PEG-DSPE-SPIONs probes by U87-MG cell were positively correlated with the co-incubation times (Figure 4A and C). The fluorescent intensity in U87-MG cells incubated with untreated nanoprobes was slightly higher than in cells incubated with serum-pretreated nanoprobes (Figure 5), consistent with NIR fluorescence imaging results (Figure 4B and D). These results demonstrated that serum protein adsorption on nanoparticles minimally impacts ligand–receptor interactions, enhancing the potential for intravenously injected nanoparticles to achieve significant tumor targeting.

### Cellular affinity and biodistribution of peptides *in vivo*

Although ^D^THR exhibited lower cellular uptake compared to THR, the result of comparative head-to-head microPET/CT imaging suggested that ^D^THR is more suitable for *in situ* applications (see Supplementary Figure S3). Following the separate injection of ^68^Ga-DOTA-^D^THR into U87-MG tumor-bearing mice and subsequent PET/CT imaging an hour post-injection, the renal bladder was identified as the primary metabolic pathway for the peptide. The incorporation of unnatural optical activity into the ^D^THR peptide significantly enhanced the stability and biodistribution of tracers make it possible to acquire clearer imaging of brain tumor.

### Construction of BBB model *in vitro*

**Figure 6.**
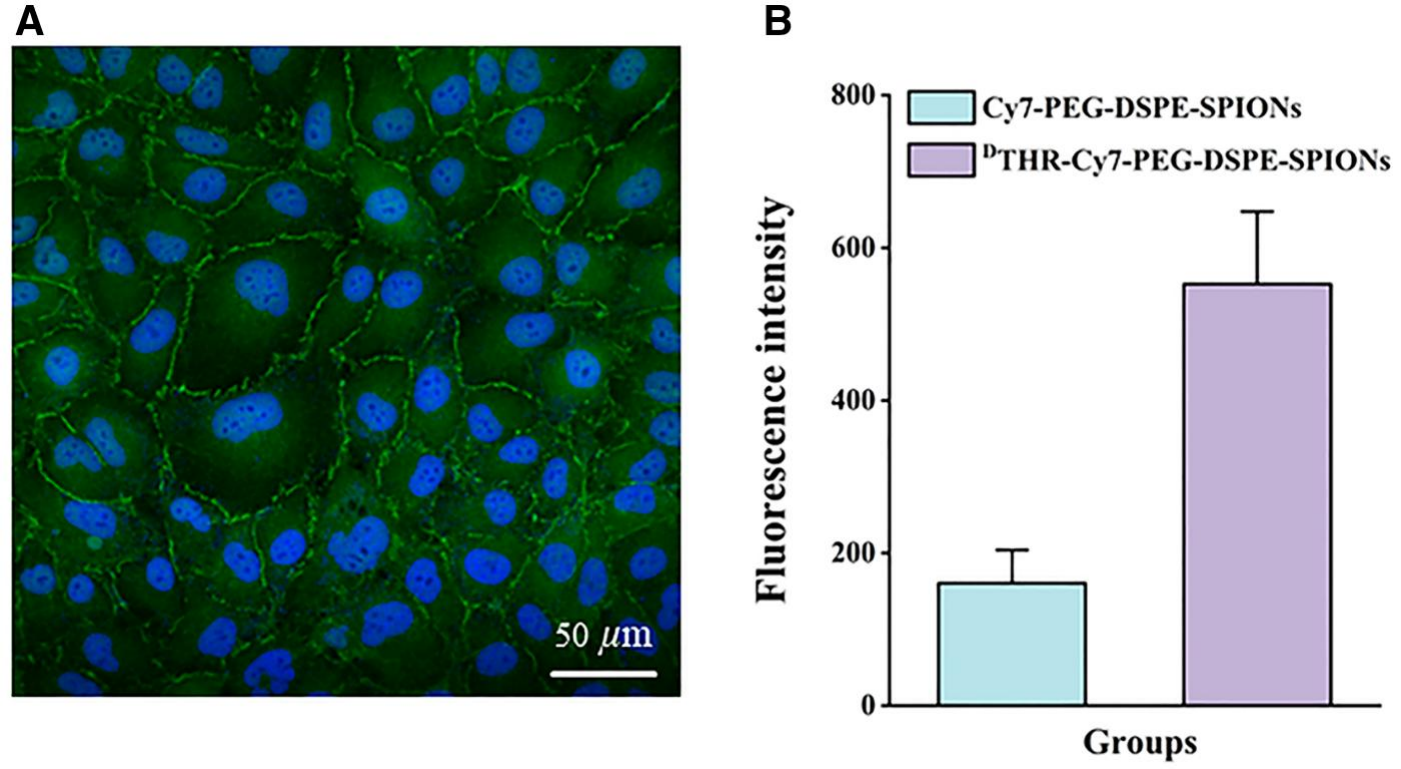
(**A**) Immunofluorescent labeling of the interstitial protein ZO-1 was performed in endothelial cells, scale bars: 50 *µ*m; (**B**) the penetrability of Cy7-PEG-DSPE-SPIONs and ^D^THR-Cy7-PEG-DSPE-SPIONs probes in the BBB model *in vitro*; the presented data represent as mean ± SEM (*n* = 3) at least three independent experiments.

To assess the functionality of BBB model *in vitro*, a series of experiments were conducted to test its permeability. First, water-leaking test conducted 7 days post-inoculation confirmed no change in liquid levels between the upper and lower chambers of the transwell, indicating a tight junction formation among endothelial cells in the BBB model. ZO-1, a tight junction protein on the endothelial cell membrane, is crucial for maintaining BBB integrity. Continuous inter-endothelial junctional staining for ZO-1 confirmed the integrity of the BBB model *in vitro* (Figure 6A). Furthermore, NaFl was used to measure the permeability [35]. And this BBB model’s Papp [(11.24 ± 0.73)×10^−6 ^cm/s] was significantly lower than that of the control group (67.80 × 10 ^−6 ^cm/s). In summary, this BBB model effectively simulates *in vitro* conditions for studying nanoprobes’ permeability across the BBB.

### Probes’ BBB penetrability *in vitro*

Fluorescence intensity of the probe solution that traversed the upper transwell chamber reflects the penetrability of each type of probe, and the results from the Cy7-PEG-DSPE-SPIONs and ^D^THR-Cy7-PEG-DSPE-SPIONs probe solutions were 158 ± 47.53 and 549 ± 97.42, respectively. The significant difference proved the superior BBB penetrability of ^D^THR-Cy7-PEG-DSPE-SPIONs probes *in vitro* (Figure 6B).

### MRI of GBM *in vivo*

**Figure 7.**
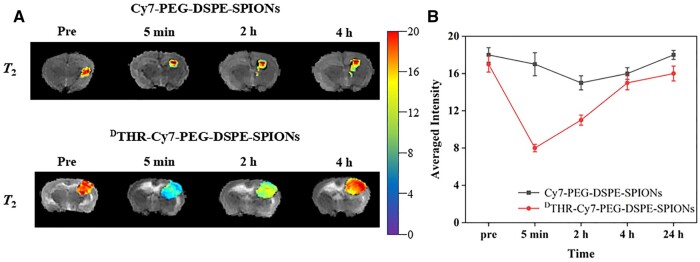
The *T_2_*-weighted MR imaging was performed on brains bearing tumors at various time points prior to and subsequent to administration of (**A**) Cy7-PEG-DSPE-SPIONs and ^D^THR-Cy7-PEG-DSPE-SPIONs probes. (**B**) The average intensity of two probes in tumor-bearing brains.

The tumor-bearing nude mice were performed *T*_2_WI scan before and after contrast agent injection, one group was Cy7-PEG-DSPE-SPIONs, another was ^D^THR-Cy7-PEG-DSPE-SPIONs group. As shown in Figure 7A, *T*_2_WI scans of the brain revealed no significant differences in signal intensity between the tumor and its surrounding normal tissue before nanoprobe administration, whereas enhanced tissue contrast was observed post-administration, due to probe accumulation in the tumor. Figure 7B effectively depicts signal values over time using a clear curve representation. In MRI enhancement, the ^D^THR-Cy7-PEG-DSPE-SPIONs group demonstrated superior effectiveness compared to the Cy7-PEG-DSPE-SPIONs group. In detail, the ^D^THR-Cy7-PEG-DSPE-SPIONs group showed higher Fe concentrations at tumor sites with more discernible and longer lasting contrast (over 24 h), while the Cy7-PEG-DSPE-SPIONs group displayed lower Fe concentrations at tumor sites with poorer contrast throughout the observation period. Evidently, ^D^THR-Cy7-PEG-DSPE-SPIONs can locate GBM more accurately and retain longer imaging capabilities owing to the superior properties of ^D^THR-mediating.

### NIR fluorescence imaging of GBM *in vitro*

**Figure 8.**
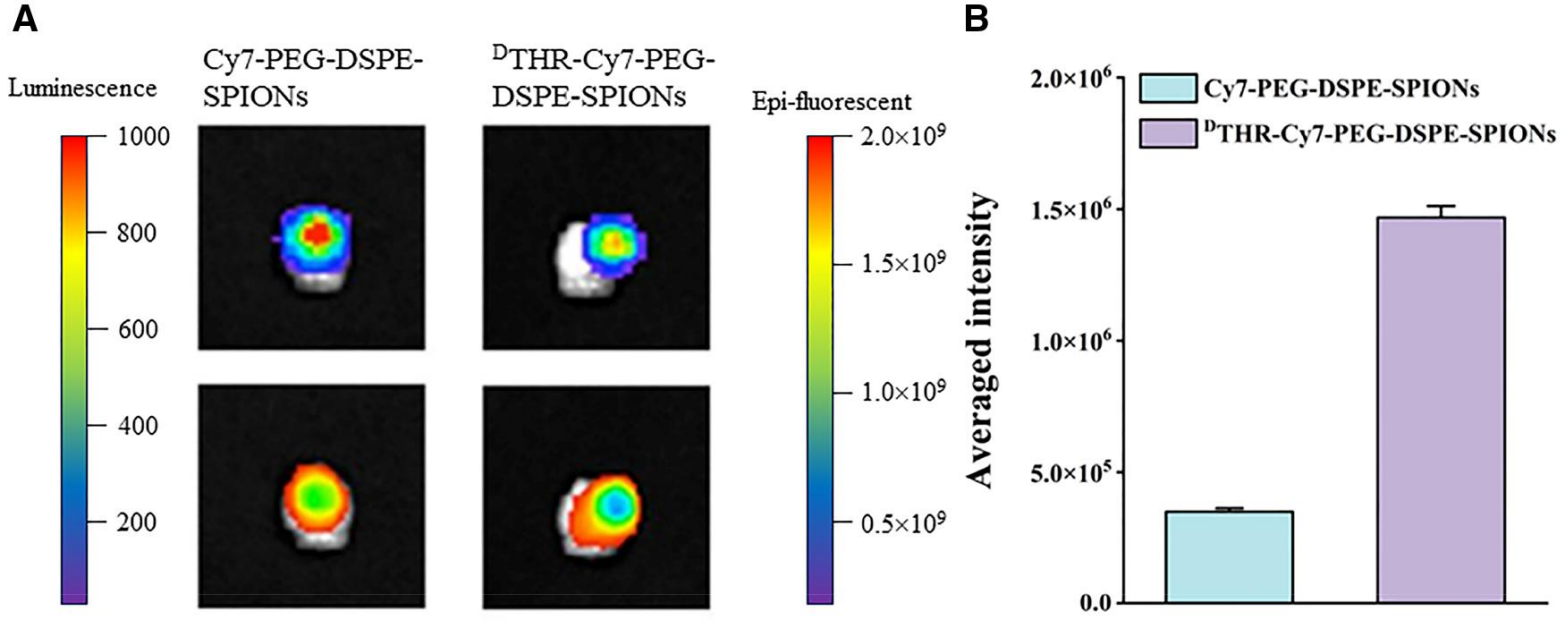
*In vitro* NIR fluorescent images of GBM tumor-bearing brains at 24 h after the intravenous injection of the nanoprobes. (**A**) The upper images are bioluminescence images showing tumor size, and the lower images are NIR fluorescent images showing probes accumulation at the tumor; and (**B**) the average intensity of two probes in tumor-bearing brains.

The mice were sacrificed 24 h post-intravenous injection of nanoprobes, and their brains were harvested to assess tumor targeting efficiency via optical imaging. The signal intensity of the tumor was measured using bioluminescence imaging and the signal intensity of the nanoprobes at the tumor site was measured using NIR fluorescence imaging. Enhanced heterogeneous fluorescent signals indicated that accumulation of both untargeted and targeted nanoprobes in the tumor’s center and margins, suggesting that ^D^THR-Cy7-PEG-DSPE-SPIONs probes accurately described the tumor margins (Figure 8A). Meanwhile, we evaluated quantitatively the targeting efficiency of nanoprobes at the GBM sites by the average fluorescence intensity (total fluorescence intensity divided by the number of bioluminescence photographs). As expected, ^D^THR-Cy7-PEG-DSPE-SPIONs showed significantly higher aggregation at GBM sites than Cy7-PEG-DSPE-SPIONs (*P *<* *0.05) (Figure 8B).

### Distribution of probes in the tumor-bearing mice

**Figure 9.**
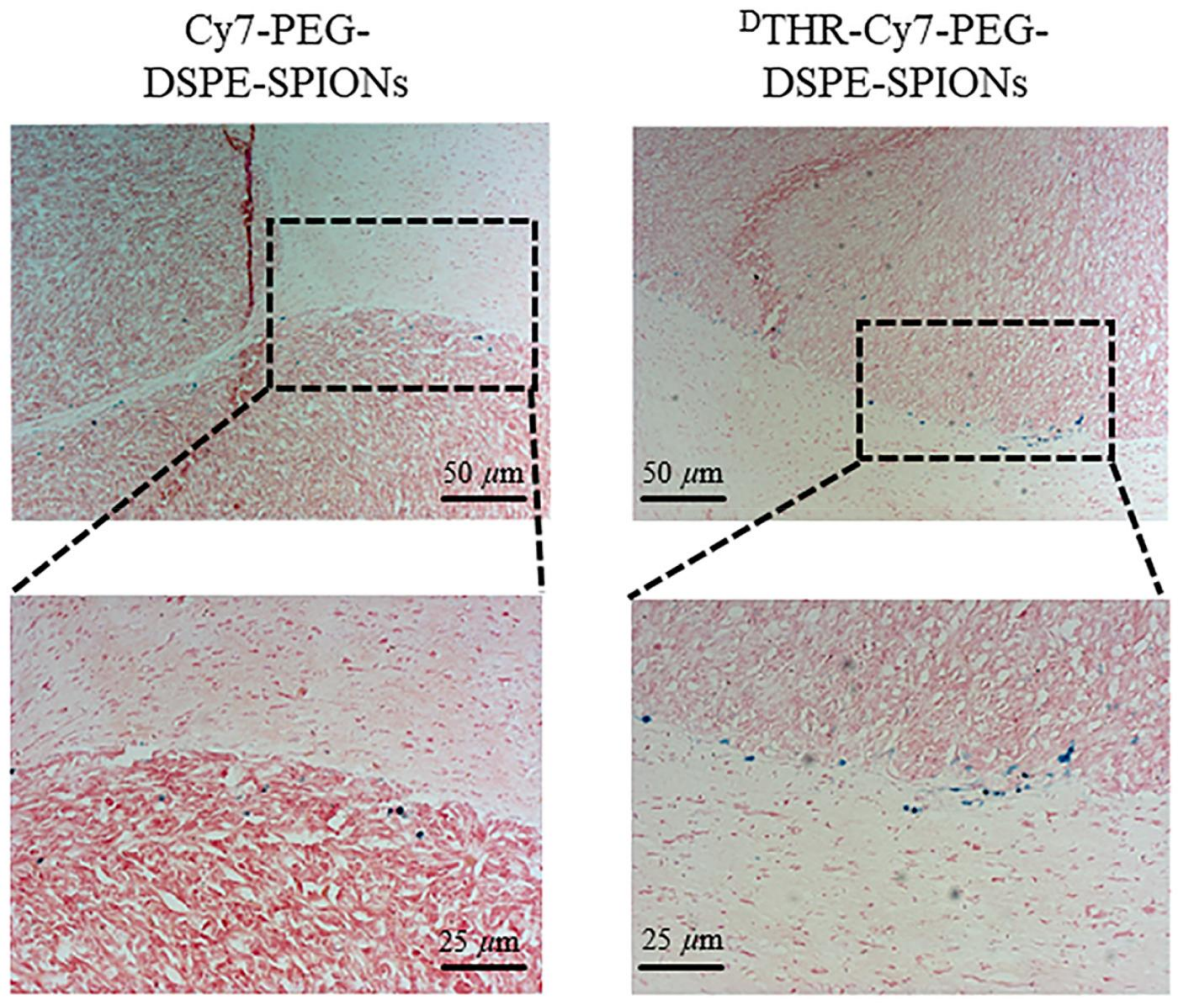
The Prussian blue staining of brain slices showed that two types of SPIONs probes aggregate at the tumor edges 24 h after the intravenous injection in tumor-bearing nude mice, the area circled by the black dotted line is tumor tissue (upper scale bars: 50 µm; lower scale bar: 25 µm).

**Figure 10.**
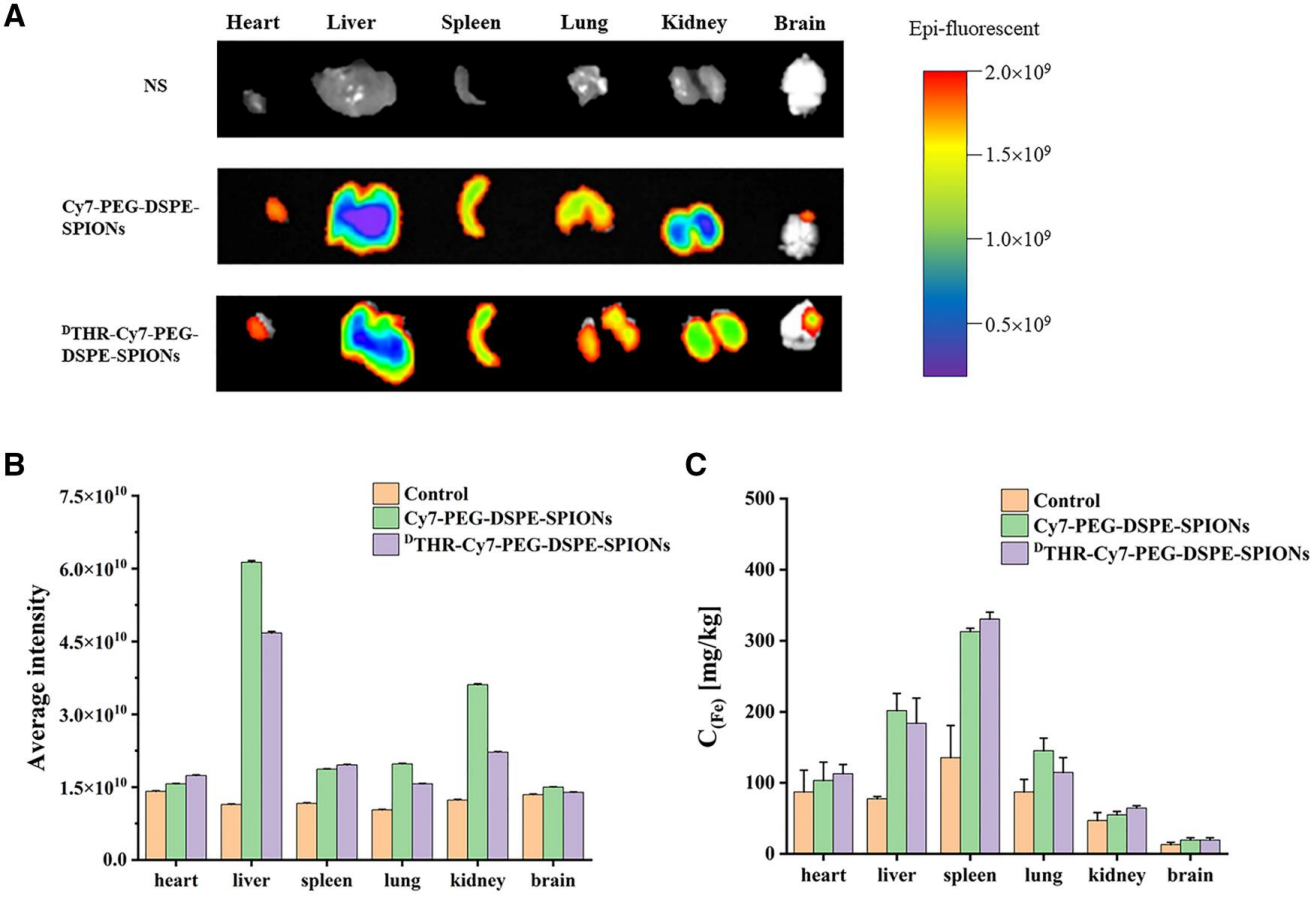
(**A**) Exemplary *ex vivo* imaging results of Cy7-PEG-DSPE-SPIONs and ^D^THR-Cy7-PEG-DSPE-SPIONs probes on tumors and major organs harvested from mice 24 h post-intravenous injection are illustrated; (**B**) the average signals of the different probes were measured by an IVIS imaging system; and (**C**) the iron content analysis of different organs from tumor-bearing mice injected intravenously with saline, Cy7-PEG-DSPE-SPIONs probes or ^D^THR-Cy7-PEG-DSPE-SPIONs probes after 24 h.

**Figure 11.**
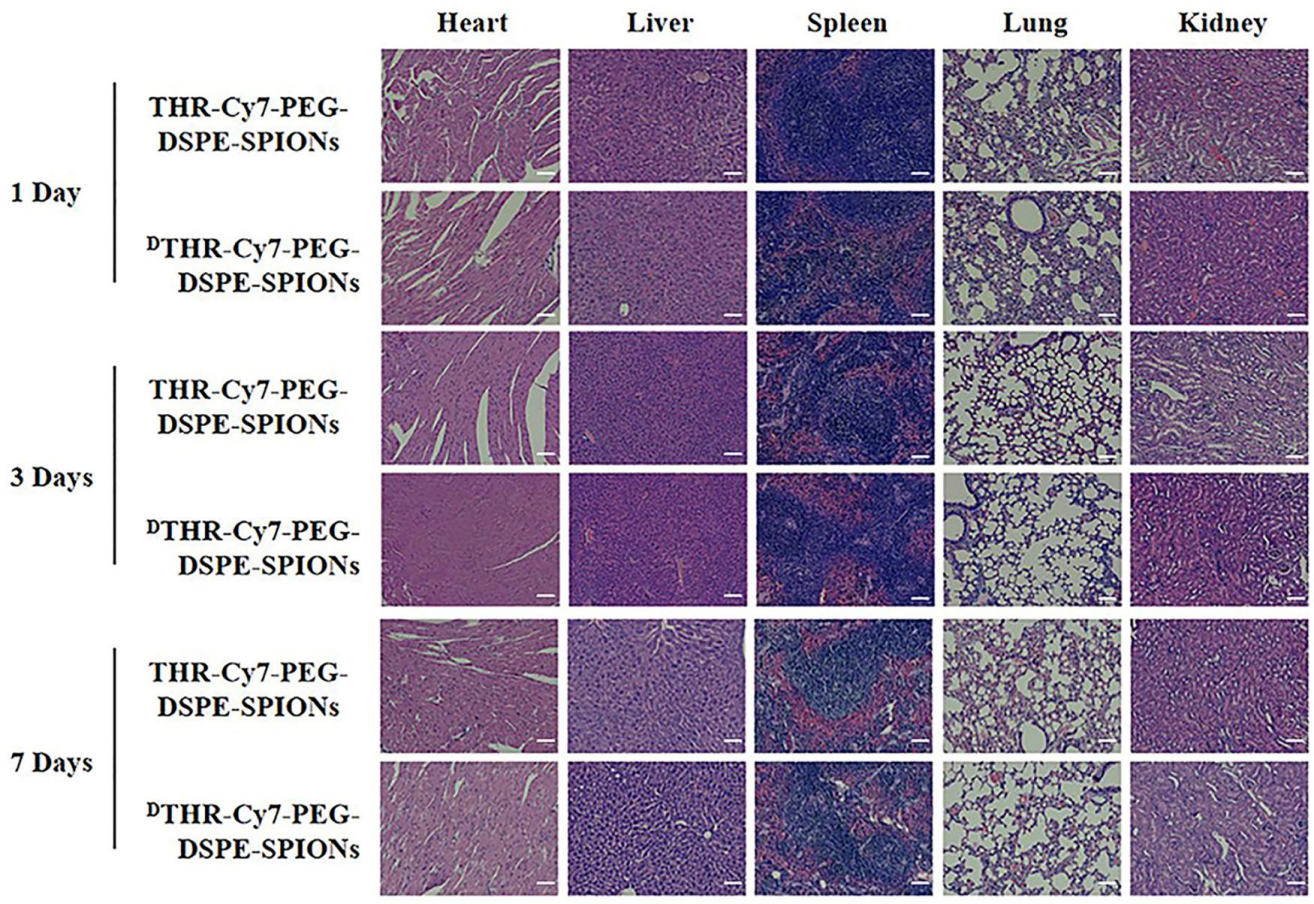
Histological changes of major organs at 1, 3 and 7 days after a single-dose intravenous injection of THR-Cy7-PEG-DSPE-SPIONs and ^D^THR-Cy7-PEG-DSPE-SPIONs probes (scale bars: 50 *µ*m).

The slices of fixed brain tissues were stained with Prussian blue, revealing patterns similar to NIR fluorescence imaging, most probes aggregated around tumor sites, with ^D^THR-modified probes showing notable higher aggregation (Figure 9). Fluorescence analysis indicated predominant nanoprobe aggregation in the spleen and liver, likely due to the non-specific uptake by the reticuloendothelial system (Figure 10A and B). In additional, rapid renal elimination of some nanoprobes was observed as expected. Fe biodistribution in tumor-bearing mice was also measured by ICP-MS 1 day post-injection. The results indicated primary accumulation of nanoprobes in the spleen and liver mainly though intravenous administration, which was consistent with the fluorescence analysis data (Figure 10C). To evaluate the toxicity of the probes *in vivo*, tail vein injection of probes (THR-Cy7-PEG- DSPE-SPIONs, ^D^THR-Cy7-PEG-DSPE-SPIONs) was administered, and H&E stained sections of major organs (heart, liver, spleen, lung and kidney) from different groups were analyzed on 1, 3 and 7 days post-injection. These analyses revealed no obvious pathological features in the experimental groups, which received different probes, compared to the control group. This indicates that the synthesized probes exhibit minimal prolonged systemic toxicity and possess favorable *in vivo* safety profiles (Figure 11).

## Conclusion

The growing application of multi-modality imaging in neuroscience has gradually attracted the attention of clinicians because its capability to overcome the limitations of a singular imaging modality, thus enhancing the sensitivity and specificity of imaging. Overall, ^D^THR-Cy7-PEG-DSPE-SPIONs nanoprobes show superior efficiency in both MRI and NIR fluorescence imaging. This efficiency is attributed to ^D^THR’s retention of THR’s primary functions and enhanced stability against proteolytic degradation in circulation, facilitating BBB crossing and GBM targeting. Meanwhile, these nanoprobes exhibit extended circulation time and demonstrate minimal biotoxicity. All the experimental results show that our peptide modification and the structure of the overall imaging agent have fully exerted their respective functions. However, ^D^THR-Cy7-PEG-DSPES-SPIONs as *T*_2_WI contrast agents have some limitations in MRI, such as non-specificity and limited contrast enhancement in certain lesions. However, their design for specific uptake by glioblastoma tissues enhances the specificity of *T*_2_WI contrast agent. As a negative contrast agent, it may reduce tissue contrast differentiation, yet it improves visualization of tissue anatomy in *T*_2_WI, which facilitates the diagnosis of diseases. On the other hand, ^D^THR-Cy7-PEG-DSPES-SPIONs effectively delineate tumor tissue boundaries as a NIR fluorescence imaging probe, their inability to penetrate the skull remains a limitation. Consequently, these novel nanoprobes show the potential to stand out in targeted GBM contrast applications, potentially making a significant contribution to the preoperative diagnosis and intraoperative localization of GBM.

## Supplementary Material

rbae015_Supplementary_Data
