# Supplementary material for: A transferrin receptor targeting dual-modal MR/NIR fluorescent imaging probe for glioblastoma diagnosis
Source: Regen Biomater. 2024 Feb 17;11:rbae015. doi: 10.1093/rb/rbae015 (PMC10939466; doi:10.1093/rb/rbae015)
Supplement: rbae015_Supplementary_Data [file rbae015_supplementary_data.docx]

Supplementary Materials for

**A transferrin receptor targeting dual-modal MR/NIR fluorescent imaging probe for glioblastoma diagnosis**

Jiaqi Hao ^1, 2, a^, Huawei Cai ^3, a^_,_ Lei Gu ^1^, Yiqi Ma ^1^, Yan Li ^1^,

Beibei Liu ^1^, Hongyan Zhu ^4^, Fanxin Zeng ^5^, Min Wu ^1, 2, *^

^1^ Department of Radiology and Huaxi MR Research Center (HMRRC), Functional and Molecular Imaging Key Laboratory of Sichuan Province, West China Hospital, Sichuan University, Chengdu, Sichuan 610041, China

^2^ Research Unit of Psychoradiology, Chinese Academy of Medical Sciences, Chengdu, Sichuan 610041, China

^3^ Department of Nuclear Medicine & Laboratory of Clinical Nuclear Medicine, West China Hospital, Sichuan University, Chengdu, Sichuan 610041, China

^4^ Laboratory of Stem Cell Biology, State Key Laboratory of Biotherapy, West China Hospital, Sichuan University, Chengdu, Sichuan 610041, China

^5^ Department of Clinical Research Center, Dazhou Central Hospital, Dazhou, Sichuan 635000, China

^a^ These two authors contributed equally.

* Corresponding author:

Min Wu, Department of Radiology and Huaxi MR Research Center (HMRRC), Functional and Molecular Imaging Key Laboratory of Sichuan Province, West China Hospital, Sichuan University, Chengdu, Sichuan 610041, China.

Tel.: +86-28-8542-2844; Fax: +86-28-8542-3503;

1. mail: wuminscu@scu.edu.cn (Min Wu).

**This PDF file includes:**

Figures 1 to 3


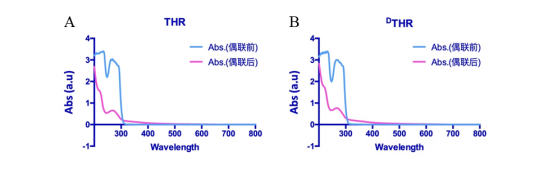


Figure S1 (a)UV–vis spectra of THR and (b) ^D^THR before and after conjugation.


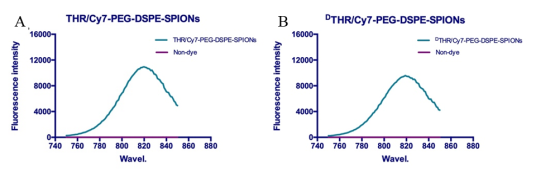


Figure S2 (a) The fluorescence intensity of THR /Cy7-PEG-DSPE-SPIONs and (b) ^D^THR /Cy7-PEG-DSPE-SPIONs probes.


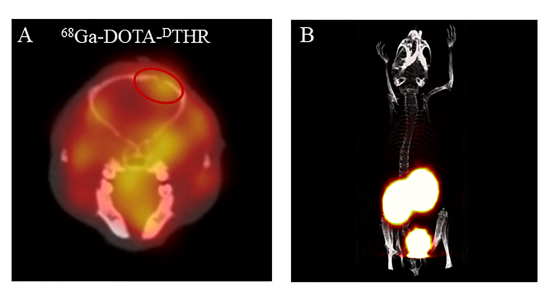


Figure S3 PET/CT scintigraph images *in vivo* distribution of probes of tumor-bearing mice. (A
